# Assessment of DNA damage with an adapted independent reaction time approach implemented in Geant4‐DNA for the simulation of diffusion‐controlled reactions between radio‐induced reactive species and a chromatin fiber

**DOI:** 10.1002/mp.14612

**Published:** 2020-12-20

**Authors:** Hoang Ngoc Tran, José Ramos‐Méndez, Wook‐Geun Shin, Yann Perrot, Bruce Faddegon, Shogo Okada, Mathieu Karamitros, Marie Davídková, Václav Štěpán, Sébastien Incerti, Carmen Villagrasa

**Affiliations:** ^1^ IRSN Institut de Radioprotection et de Sûreté Nucléaire BP17 Fontenay aux Roses 92262 France; ^2^ Department of Radiation Oncology University of California San Francisco San Francisco CA 94115 USA; ^3^ Université de Bordeaux CNRS/IN2P3 UMR5797 Centre d’Études Nucléaires de Bordeaux Gradignan Gradignan 33175 France; ^4^ Department of Radiation Convergence Engineering Yonsei University Wonju 26493 Korea; ^5^ KEK 1‐1, Oho Tsukuba Ibaraki 305‐0801 Japan; ^6^ Radiation Laboratory University of Notre Dame Notre Dame In 46556 USA; ^7^ Department of Radiation Dosimetry Nuclear Physics Institute of the CAS Prague Czech Republic

**Keywords:** diffusion‐controlled reaction, DNA damage, Geant4‐DNA, IRT

## Abstract

**Purpose:**

Simulation of indirect damage originating from the attack of free radical species produced by ionizing radiation on biological molecules based on the independent pair approximation is investigated in this work. In addition, a new approach, relying on the independent pair approximation that is at the origin of the independent reaction time (IRT) method, is proposed in the chemical stage of Geant4‐DNA.

**Methods:**

This new approach has been designed to respect the current Geant4‐DNA chemistry framework while proposing a variant IRT method. Based on the synchronous algorithm, this implementation allows us to access the information concerning the position of radicals and may make it more convenient for biological damage simulations. Estimates of the evolution of free species as well as biological hits in a segment of DNA chromatin fiber in Geant4‐DNA were compared for the dynamic time step approach of the step‐by‐step (SBS) method, currently used in Geant4‐DNA, and this newly implemented IRT.

**Results:**

Results show a gain in computation time of a factor of 30 for high LET particle tracks with a better than 10% agreement on the number of DNA hits between the value obtained with the IRT method as implemented in this work and the SBS method currently available in Geant4‐DNA.

**Conclusion:**

Offering in Geant4‐DNA more efficient methods for the chemical step based on the IRT method is a task in progress. For the calculation of biological damage, information on the position of chemical species is a crucial point. This can be achieved using the method presented in this paper.

## 1 INTRODUCTION

Among the possible causes of the effects induced by ionizing radiation on living organisms, DNA damage is of particular interest^1^ and many techniques are being developed to both measure and predict it. The creation of radiolytic species through ionization and excitation in the vicinity of the biological target significantly contributes via the so‐called indirect damage. Investigations of indirect damage consider the mechanisms of free radical attack on the DNA target, including taking account of its chemical and geometrical structure. The accuracy of the assessment of indirect damage depends in part on the knowledge of the positions of radiolytic species in relation to the DNA molecule. This depends on the ability of each of these species to diffuse and react with other molecules in the environment, including DNA. For modeling purposes, the basic DNA elements (2‐deoxyribose, phosphate, base) are in Geant4 often represented by static reactive spheres (or sinks) linked together to form more complex structures, from the nucleotide pair to the genome.^2^, ^3^, ^4^, ^5^, ^6^ The reaction mechanism is diffusion‐controlled, as it is supposed to be,^7^ and triggered when radiolytic species diffuse and encounter a reactive site (generally reactive spheres) that are either representing the sugar‐phosphate backbone or the bases of a nucleotide. This simple model still ignores the overlap of the reaction sites as well as the overlap of multiple reactive centers in the sugar‐base system.^8^ However, it is well suited when focusing on the assessment of the DNA damage on nucleotide or base‐pair level.

The theory of diffusion‐controlled reactions^9^ describes solutions of the diffusion equation (Smoluchowski equation) with boundary conditions. Based on these solutions, stochastic simulation techniques, through step‐by‐step (SBS) or independent time reaction (IRT) methods, describe that the diffusion of molecules and the reactions between reactants^10^ are used to simulate the evolution of heterogeneous reactive species distributions from the initial radiolysis. The SBS method^11^, ^12^ is able to provide the spatial positions of the diffusing species at a given time. This knowledge of the temporal evolution of trajectories calculated in discretized time steps may be advantageous for the assessment of indirect DNA damage, as discussed in Section 3. The accuracy of this method depends on the determination of the time step, requiring a compromise between accuracy, which makes necessary a sufficiently small time step, and calculation time, which increases dramatically with a decreasing time step. Indeed, the SBS method shows very long computation times for radiobiology applications. In that frame, IRT method has been developed to save a considerable amount of computing time. However, this method does not provide exact positions of the radicals in time.^10^ Indeed, the IRT method is based on the “Independent Pair Approximation”; thus, reactive pairs are assumed independent, that is, the reaction time between two reactants does not depend on the other reactants present in the medium, and the diffusion time can be calculated directly using Green’s functions.^13^, ^14^ The predictions of the IRT method agree with SBS for systems of two particles in diffusive motion for totally and partially diffusion‐controlled reactions.^10^, ^15^

An important question is how the “Independent Pair Approximation” method could be applied to reactions of radicals with biological molecules which are considered static in the simulated environment. Based on a simple model of biological system as DNA elements, which are represented by two reactive spheres linked together at a fixed distance, Bluett and Green^8^ have reported the competitive effect as a function of the intersphere distance of reaction probability of diffusion‐influenced reactions and found an overestimation of this probability with the IRT method compared to an exact analytical solution. Due to the complexity of many reactive sites in a complex DNA geometry model, solving the problem as an exact solution is intractable. In this work, an IRT method is used in Monte Carlo simulation of free radical attacks on DNA.

Among the Monte Carlo codes, Geant4^16^, ^17^, ^18^ is a general purpose toolkit first dedicated to high energy and nuclear physics. Developments have enabled other applications in medical or space fields. Geant4‐DNA^19^, ^20^, ^21^, ^22^ is an extension of Geant4 for the modeling of biological damage induced by ionizing radiation at the nanometer scale. To complement comparisons of our results, we have used published data obtained with TOPAS‐nBio.^23^ TOPAS is a Monte Carlo^24^ system that wraps and extends Geant4 to facilitate the use of Monte Carlo simulation of radiotherapy by medical physicists. TOPAS‐nBio is the extension of TOPAS to model radiobiological at the cellular and subcellular scale. Since the release of Geant4 version 10.1, Geant4‐DNA provides a computational framework for the physicochemical and chemical stage in which the SBS method, that includes Brownian motion and chemical reactions between molecules resulting from water radiolysis, is available for users.^11^ Applications are mainly related to the validation of radiolytic yields and the evaluation of radiation‐induced DNA damage. To enhance the potential of Geant4‐DNA, developments are ongoing in different groups with the goal to provide IRT methods to Geant4‐DNA users.^25^, ^26^ In particular, an implementation of the original IRT method has been developed and will be released soon.^25^ In this work, an implementation of an IRT variant is proposed. This method is implemented in the current chemistry module of Geant4‐DNA with the idea of keeping the spatiotemporal information of the radiolytic species to calculate DNA damage. The proposed method brings the possibility to choose a reasonable compromise between the gain in calculation time over the SBS method and the accuracy of the assessment of the DNA damage using a molecular geometrical model of a segment of chromatin fiber. To do so, this new IRT method is complemented with the synchronous algorithm described in Karamitros et al.^11^ Prior to the public release of this method, this implementation is compared to the current SBS technique, and the differences are assessed in terms of calculation time and quantities of interest. The radiochemical yields calculated with our IRT method are compared to those obtained with the SBS method of Geant4‐DNA, to those obtained with TOPAS‐nBio, using the IRT method described in Schuemann et al.,^23^ and to published experimental data. Finally, a DNA reaction model of free radicals based on reactive sinks of a heterochromatin fiber geometry is used to compare DNA damage calculated in Geant4‐DNA using the newly implemented IRT method and the existing SBS method.

## 2 MATERIALS AND METHODS

### 2.A Diffusion‐controlled reactions

The reactions of radicals with each other or with DNA molecules can be described as diffusion‐controlled reactions.^27^ Diffusion‐controlled reactions are reactions occurring immediately when molecules encounter each other. The reaction rate is equal to the rate of diffusion of the reactants through the medium when the reaction is totally diffusion‐controlled and takes into account the steady‐state rate constant from the encounter to their reaction when the reaction is partially diffusion‐controlled. In this section, we briefly present the probability distribution of these reactions and sampling techniques for an independent two‐body system.

#### 2.A.1 Totally diffusion‐controlled reaction

Suppose that the two reactants considered are labeled 1 and 2. The solution of the Smoluchowski equation with an absorption boundary condition (so‐called totally diffusion‐controlled reaction) transformed to radial Green's function in a spherical coordinate system is used to deduce the probability of reaction $p(t|r_{0})$ as Ref. [13]:

(1) $$p\left(t|r_{0}\right)=\frac{R}{r_{0}}erfc\left[\frac{r_{0}‐R}{\sqrt{4Dt}}\right]$$

where R is the reaction radius which is sum of reaction radius of both reactants 1 and 2, $r_{0}$ is the initial distance between reactants 1 and 2, D is the relative diffusion coefficient and equals $D_{1}+D_{2}$ where *D*_1_ and *D*_2_ are the diffusion coefficients of reactant 1 and 2, respectively. The experimental reaction rate $k_{\mathrm{obs}}$ is used to define R by the relation $k_{\mathrm{obs}}=4\pi RD$.

This probability can be used for charged species by replacing effective distances $d_{\mathrm{eff}}$ to $r_{0}$:

$$d_{\mathrm{eff}}=‐\frac{r_{c}}{1‐e^{\left(\frac{r_{c}}{d}\right)}}$$

where $r_{c}$ is the Onsager radius.^13^ In this work, $r_{c}=0.71\;\text{nm}$ (particles with charge equal to 1, in liquid water at 25°C).

#### 2.A.2 Partially diffusion‐controlled reaction

In the radiation boundary condition (or partially diffusion‐controlled reaction), Smoluchowski equation gives a well‐known distribution for calculating the distribution probability:

(2) $$p\left(t|r_{0}\right)=P_{0}\left(erfc\left(y\right)‐{\;e}^{\left(x^{2}+2xy\right)}erfc\left(x+y\right)\right)$$

where $P_{0}=\frac{R_{\mathrm{eff}}}{r_{0}}$, $R_{\mathrm{eff}}=R\frac{k_{\mathrm{act}}}{k_{\mathrm{dif}}+k_{\mathrm{act}}}$, $x=\frac{\left(k_{\mathrm{dif}}+k_{\mathrm{act}}\right)\sqrt{\mathrm{Dt}}}{k_{\mathrm{dif}}R}$ and $y=\frac{r‐R}{\sqrt{4Dt}}$, $k_{\mathrm{dif}}=4\pi RD$ is the reaction rate constant of transport “to encounter” and $k_{\mathrm{act}}=4\pi R^{2}v$ is the steady‐state rate constant “from encounter” to the reaction which can be defined by the velocity of reaction v but usually calculated using the experimental reaction rate $k_{\mathrm{obs}}$ in relation:

(3) $$\frac{1}{k_{\mathrm{obs}}}=\frac{1}{k_{\mathrm{dif}}}+\frac{1}{k_{\mathrm{act}}}$$

For charged species, x and y can be calculated as follows^13^:

(4) $$x=\frac{4R^{2}\alpha^{\prime }}{r_{c}^{2}}\sqrt{A}\frac{t}{D}\sin h^{2}\left(\frac{r_{c}}{2R}\right),$$

and

(5) $$y=\frac{r_{c}\left[coth\left(\frac{r_{c}}{2r_{0}}\right)‐coth\left(\frac{r_{c}}{2R}\right)\right]}{2\sqrt{4Dt}},$$

where

(6) $$\alpha^{\prime }=v+\frac{r_{c}D}{R^{2}\left[1‐e^{\left(‐\frac{r_{c}}{R}\right)}\right]},$$

In this case,

(7) $$R_{\mathrm{eff}}=\frac{‐r_{c}}{1‐\left[1+Dr_{c}/vR^{2}\right]e^{\left(r_{c}/R\right)}},$$

but practically is calculated from the experimental reaction rate $k_{\mathrm{obs}}$ with the relation $k_{\mathrm{obs}}=4\pi R_{\mathrm{eff}}D$.

##### SBS method

One of the methods that can be implemented for the simulation of diffusion‐controlled reactions is SBS method. A detailed description of the SBS model using dynamic time step implemented in Geant4‐DNA can be found in Karamitros et al.^11^ Briefly, this method proposes a time step model that allows the choice of virtual time steps during which the reaction cannot occur with at least 95% (by default) confidence (named dynamic time step). One can visualize this as creating a protection domain that surrounds the particle, ensuring that this particle will not react with any other particle up to its border with 95% confidence. Therefore, in the protection domain, the particle is considered approximately independent and may be able to take longer diffusion steps. The function in Eq. (1) is used to evaluate this time step. This process is repeated many times until a chemical interaction takes place. Thus, we may have one or many time steps before the reaction occurs. To avoid the scenario of many small time steps, the *Minimum Time Steps* and the Brownian bridge technique have been added to limit the number of time steps to an encounter. While *Minimum Time Steps* constrain the minimum time step allowed for each reactant pair, the Brownian bridge technique computes the probability of encounter during their *Minimum Time Steps* and thus compensates for the “missed” reactions. Detail of these techniques can be found elsewhere.^10^, ^11^

To save computation time, it can be decided to increase the *Minimum Time Step* value as function of the virtual time used in the simulation using the predefined static time steps proposed by Kreipl et al.,^28^ since radical spurs become more and more sparse. This is the *SBS‐dynamic time steps* using variable *Minimum Time Step* values. It is also important to note that the current SBS‐*dynamic time step* method assumes that all reactions are totally diffusion‐controlled.

##### IRT method

IRT is another method for simulating diffusion‐controlled reactions. By simplifying the multiple particle problem to the two‐particle problem in an approximation, the IRT method is based on the relative comparison of random times to reaction calculated for all possible reactant pairs independently of the particle system. The minimum reaction time obtained is selected such that the corresponding reaction will occur in the next step. These random times are sampled from the reaction probability distributions of the reactant pairs which are considered independent of each other and in infinite space. Therefore, only the initial spatial distribution of reactant pairs plays an important role on these probability distributions.

The IRT method determines the minimum time to the next reaction. Reactive products created by reactions that have occurred can undergo reactions with other reactants. These new reactions need to be considered and included in the possible reaction times, depending on their initial positions. Based on an approximation proposed by Clifford et al.,^10^ models for reactive products allow to calculate their reaction times. We briefly summarize these models as follows:

- *Diffusion and time approaches*: These approaches propose the deduction of the reaction time of reactive products based on their parent pair position and the difference of diffusion coefficient between them and their parents. While the time approach uses a rescaling of the reaction time of parent reactant pair to calculate the reaction time of products, the diffusion approach recalculates the random time corresponding to the distance between products and their reactants. Thus, the position of reactants is not determined.
- *Position approach*: In this approach, the minimum required time for one possible reaction in the system is considered as a “time step.” In the meantime, all remaining reactive radicals are diffused in a single step to this time step following a Gaussian distribution of their positions as Brownian objects. In this approach, the position of reactive products following the reactions are determined explicitly and the reaction times between the products and all the remaining radicals involving the products are simulated.

### 2.B IRT method proposed in this work

#### 2.B.1 Synchronous event‐driven algorithm

As explained in the introduction, in the traditional IRT method,^10^, ^13^ due to the fact that space is assumed infinite, only relative distances of these reactive pairs are considered to calculate their reaction probability. Under this assumption, IRT is an exact method for two diffusive particle systems in an infinite and homogeneous space.^8^ Based on this assumption, the reaction pairs are simulated independently and asynchronously in time and space. While this is a considerable advantage in terms of computing time, the spatial–temporal information of the system is not simulated explicitly. As a complementary approach, our particular implementation of the IRT method is designed to be adapted to the chemical module of Geant4‐DNA. This implementation uses the sampled random time given by the reaction probabilities in Section 2.A [by using Eqs. (1) and (2)] as a function of time step (or time slice) to the next reaction that should occur. In other words, instead of optimizing the time step to the next reaction as Geant4‐DNA SBS‐dynamic time step method does, we calculate directly the time to the reaction of an independent reactive pair. Therefore, each step of the simulation consists of two stages: (a). the reaction times of all possible reactant pairs are calculated; (b). the minimum reaction time and corresponding reaction are selected to trigger. The reactive product positions created in the reactions and the remaining molecules are considered explicitly together to diffuse for the time step using the position approach (see Section 2.A.2). Then, based on their new positions, the new random reaction times are re‐evaluated sequentially for all the radicals in the system and the new minimum reaction time and corresponding reaction is selected for next time step. This procedure is repeated until the end time of simulation. Consequently, for each time step, we need to update the time and position of all molecules in diffusion. This is the main drawback of this approach. However, this provides explicitly spatiotemporal information of the reactive species after each time step which can then be coupled with information on the geometrical boundaries or the biological target.

When reactions with DNA or other biomolecules are considered, the reaction times are compared for each reactive pair, keeping the spirit of independent pair approximation. The resulting minimum reaction time is then compared with the minimum reaction time between radicals themselves. The smaller of the times will define the type of reaction that will occur: radical/radical (Table I) or radical/DNA (Table III).

**Table I:** Reactions and reaction rate coefficients used in this work. For IRT, the list of reactions are assigned by type: partially diffusion‐controlled, totally diffusion‐controlled, and spin statistical factor.^30^ For SBS, all the reactions in the table are considered totally diffusion‐controlled reactions.

Reaction	$k_{\mathrm{obs}}$ (×10^10^ M^−1^s^−1^)	Partially diffusion‐controlled	Totally diffusion‐controlled	Spin statistical factor
H^•^ + $e_{\text{aq}}^{‐}$ + H_2_O → OH^−^ + H_2_	2.5		X	X
H^•^ + ^•^OH → H_2_O	1.55	X		
H^•^ + H^•^ → H_2_	0.503		X	X
H_2_O_2_ + $e_{\text{aq}}^{‐}$ → OH^−^ + ^•^OH	1.1	X		
H_3_O^+^ + $e_{\text{aq}}^{‐}$ → H^•^ + H_2_O	2.11	X		
H_3_O^+^ + OH^−^ → 2H_2_O	11.3		X	
^•^OH + $e_{\text{aq}}^{‐}$ → OH^−^	2.95	X		
^•^OH + ^•^OH → H_2_O_2_	0.55	X		
$e_{\text{aq}}^{‐}$ + $e_{\text{aq}}^{‐}$ + 2H_2_O → 2OH^−^ + H_2_	0.636		X	X

It is worth noting that due to the free diffusion of all remaining radicals for each time step, the inter‐reactant distance of some reactant pairs may be smaller than the reaction radius and they may cause extra reactions with the probability of reaction^29^:

(8) $$℘=\frac{e^{\left(‐\frac{r_{c}}{R}\right)}‐e^{\left(‐\frac{r_{c}}{R+R_{s}}\right)}}{e^{\left(‐\frac{r_{c}}{R}\right)}‐e^{\left(‐\frac{r_{c}}{R+R_{s}}\right)}‐\left(\frac{k_{\mathrm{dif}}}{k_{\mathrm{act}}}\right)\left[1‐e^{\left(‐\frac{r_{c}}{R}\right)}\right]},$$

For radicals without charge, ℘ is:

(9) $$℘=\frac{R_{s}}{R_{s}+\left(k_{\mathrm{dif}}/k_{\mathrm{act}}\right)\left(R+R_{s}\right)},$$

The interpretation may be addressed by an unsuccessful encounter where $R_{s}$ is the separation distance of the encounter, equals 0.3 nm.

For totally diffusion‐controlled reactions for which ($k_{\mathrm{act}}\to \infty$), ℘=1, the reaction immediately occurs. Moreover, a spin statistical factor for any encounter of *H^•^* and $e_{\mathrm{aq}}^{‐}$ or their combinations (see Table I) is added to consider the fact that only the singlet configuration of their spins allows the occurrence of the reaction.^14^

#### 2.B.2 Sampling method for partially diffusion‐controlled reactions

Following the description of Green et al.,^13^ the IRT method requires the generation of random times from these reaction probability distributions (see Section 2.A) for each of the reactant pairs at any initial distance between them. For total diffusion‐controlled reactions, the inversion method^13^ provides a simple way of calculating these reaction times by using the inverse of the error function in Eq. (1). The same method is difficult to apply when considering partially diffusion‐controlled reactions [Eq. (2)], because of the complexity of this equation. In this case, to sample time *t*, a technique previously proposed by Bluett and Green^8^ can be applied, in which an imaginary trajectory of one reactant (particle 1 which is considered moving) from the initial position to the reaction point with other reactant (particle 2 which is considered not moving) is split into two parts of the time step. The first part of time step corresponds to the time spent from the initial position of reactant 1 *to encounter* at the reaction radius *R*. This time step can be sampled using the distribution of Eq. (1) as a totally diffusion‐controlled reaction. After the encounter, the reactant is assumed to be standing at the boundary of the reaction radius where $r_{0}=R$ and is substituted in Eq. (2):

(10) $$p\left(t|R\right)=\frac{k_{\mathrm{act}}}{k_{\mathrm{dif}}+k_{\mathrm{act}}}\left(1‐e^{x^{2}}erfc\left(x\right)\right)$$

The second part of the time step corresponds to the time between the first encounter to the final reaction between both reactants and is sampled from this distribution [Eq. (10)]. We summarized the second sampling as follows^8^:

1. Generate two uniform [0,1] random numbers $U_{1}$ and $U_{2}$
2. If $U_{1}$ is greater than $\frac{k_{\mathrm{act}}}{k_{\mathrm{dif}}+k_{\mathrm{act}}}$, then the reaction will not take place
3. Otherwise, generate an absolute value of normally distributed random Y with mean 0 and standard deviation $\sqrt{2}$
4. Calculate $t=\left\{\frac{1}{D}{\left(‐\frac{Rk_{\mathrm{dif}}\ln\left(U_{2}\right)}{\left(k_{\mathrm{dif}}+k_{\mathrm{act}}\right)Y}\right)}^{2}\right\}$ as a random time for the second sampling.

**Fig. 1:**
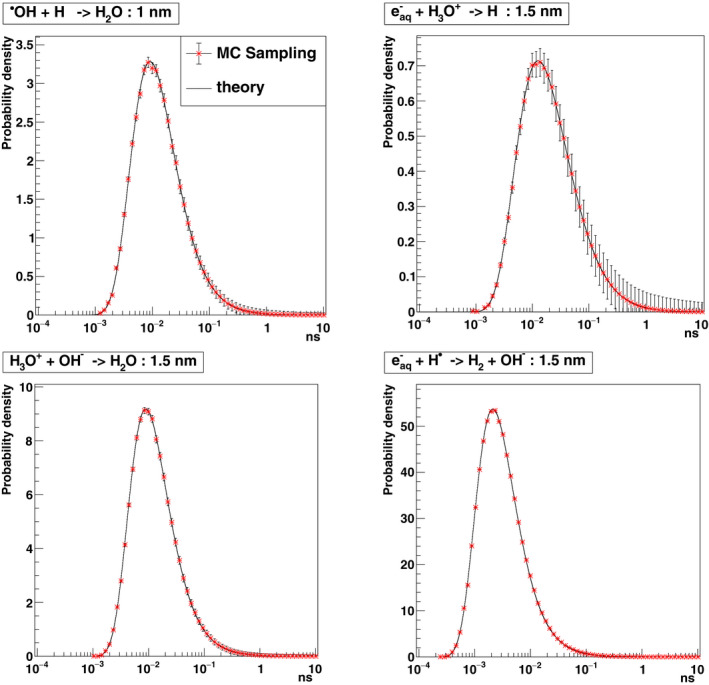
Sampled times to reaction from the probability densities of reactions (“theory”)^30^ using the IRT method presented in this work. H^•^ + ^•^OH → H_2_O (initial distance of reactants 1.0 nm, partially diffusion‐controlled), H_3_O^+^ + $e_{\text{aq}}^{‐}$ → H^•^ + H_2_O (initial distance of reactants 1.5 nm, partially diffusion‐controlled), H_3_O^+^ + OH^−^ → 2 H_2_O (initial distance of reactants 1.5 nm, totally diffusion‐controlled), H^•^ + $e_{\text{aq}}^{‐}$ + H_2_O → OH^−^ + H_2_ (initial distance of reactants 1.5 nm, totally diffusion‐controlled).

The reaction time is the sum of times of two parts of the time step. Figure 1 presents the sampled times using the sampling algorithm for four reactions among those shown in Table I combining both totally and partially diffusion‐controlled reactions. These four reactions are sampled using 10^6^ histories with an initial distance in this example being 1.0 or 1.5 nm for each reactant pair. In all cases, the sampling distributions are as predicted by the probability density functions that are mentioned by Ref. [30]. The error bars in the figure show the square of weight deviation for each point.

### 2.C IRT and SBS reaction schemes

Two reaction schemes for IRT were studied in this work. The first scheme combined reactions and reaction rate coefficients for partially diffusion‐controlled, totally diffusion‐controlled and spin statistical factor correction (Table I) and indicated as “this work” in the results section. The second scheme only considered totally diffusion‐controlled reactions, indicated as “this work‐TDC” in the results section. This second scheme had been considered for a “fair” comparison with the SBS method, currently available in Geant4‐DNA, where all the reactions shown in Table I are handled as totally diffusion‐controlled.

### 2.D Description of the geometrical models and simulation setups

The time evolution of the number of molecular species created or lost per 100 eV of deposited energy (G‐value) was obtained with the different reaction schemes of Section 2.B. The simulation setup consisted of a box of homogeneous liquid water (1 g/cm^3^) with dimensions representing a “pseudo infinite” region (cube of 1 km side). For computational time and memory reasons, the particle source consisted of 80 keV electrons shot from the center of the box. The initial chemical species are obtained from physical track interactions for each primary electron considered independently of each other and depositing a total energy of 1 keV using the physics list G4EmDNAPhysics_option8 in order to facilitate comparisons with previously published results from TOPAS‐nBio.^23^ The corresponding process classes, model classes, and energy ranges of this physics list for electrons and protons are shown in Table II. It is equivalent to the physics list used by default except for the elastic scattering of electrons where the CPA100 code model is used for energies below 256 keV. More details can be found elsewhere.^31^ The temporal evolution of G‐values is recorded over the time range from 1 ps to 1 μs.

**Table II:** Content of the physics list G4EmDNAPhysics_option8 used in this study : processes, models, and energy ranges for electrons and protons.

Particle	Process	Model	Energy range
Electron	G4DNAElastic	G4DNACPA100ElasticModel	11 eV–256 keV
		G4DNAChampionElasticModel	256 keV–1 MeV
	G4DNAExcitation	G4DNABornExcitationModel	9 eV–1 MeV
	G4DNAIonisation	G4DNABornIonisationModel	11 eV–1 MeV
	G4DNAVibExcitation	G4DNASancheExcitationModel	2 eV–100 eV
	G4DNAAttachment	G4DNAMeltonAttachmentModel	4 eV–13 eV
Proton	G4DNAElastic	G4DNAIonElasticModel	100 eV–1 MeV
	G4DNAExcitation	G4DNAMillerGreenExcitationModel	10 eV–500 keV
		G4DNABornExcitationModel	500 keV–100 MeV
	G4DNAIonisation	G4DNARuddIonisationModel	0 keV–500 keV
		G4DNABornIonisationModel	500 keV–100 MeV
	G4DNAChargeDecrease	G4DNADingfelderChargeDecreaseModel	100 eV–100 MeV

In a practical way, in next releases we will modify the recent Geant4‐DNA exemple dnadamage1 in order to offer the possibility to use both chemistry approaches: the current SBS method and the new IRT method presented in this work and this only by changing the chemistry list. More details about the C++ classes and their structure related to this method will be given to the users through the README file of the example.

**Fig. 2:**
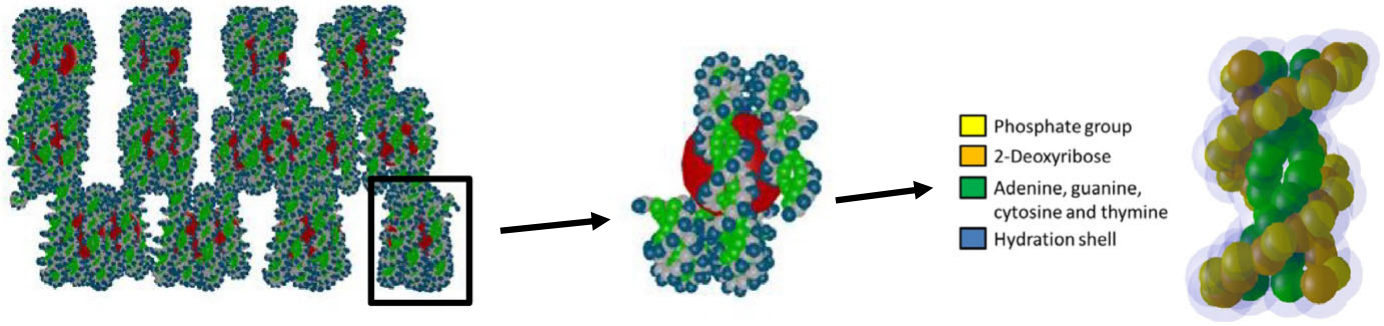
40 nm heterochromatin fiber (including in 18 nucleosomes and 19 linkers for a total of 3640 nucleotide pairs) in a cubic voxel of 40 nm generated with the DNAFabric tool.^2^

In that example, as well as in this work, the DNA model was generated with the DNAFabric tool.^6^ DNAFabric is a stand‐alone software which enables complex 3D geometries to be generated and visualized, in particular DNA structures from the nucleotide constituents (2‐deoxyribose, phosphate, and DNA bases) to the whole genome representation. The geometrical model generated consisted of a piece of a 40 nm heterochromatin fiber including 3640 nucleotide pairs in a cubic voxel of 40 nm centered in a box of 2 µm side made of liquid water (1 g/cm^3^). These DNA elements representing the 2‐deoxyribose, phosphate, and DNA bases (adenine, guanine, cytosine, and thymine) were considered as static spherical traps. Using Protein Data Bank information,^32^ atom's volumes and positions of each of these molecules were assembled in a unique sphere. From these spheres, nucleotide pairs were constituted and served as the basic units forming a double helix of the *B‐DNA* configuration. More information about the generation of this geometrical model can be found elsewhere.^2^, ^6^ Some segments of this double helix were then twisted around histone proteins represented by a sphere acting like a histone with a 2.4‐nm radius, building a nucleosome.^33^ After that, nucleosomes were linked together to create the chromatin fiber (see Fig. 2). Additionally, a volume corresponding to water layers wrapped around each of the DNA elements was modeled as an outer hydration shell. This geometry can also integrate histones whose scavenging capacity would be simulated by introducing a specific reaction. Nevertheless, in this study, histones were not considered in order to maximize the probability of interaction and reaction with the DNA geometry.

In order to maximize this probability as well, the simulation was performed using 500 keV protons of normal incidence with respect to a side of the voxel and homogeneously distributed in a circle of radius 20 nm. To compute DNA damage, we used the criteria presented elsewhere.^2^, ^33^ In short, the total number of damages was computed as the sum of direct and indirect damage. A direct damage is scored if the cumulative deposited energy from ionizations and excitations in the individual volumes of a nucleotide backbone (i.e., the volumes representing a group of the phosphate, the 2‐deoxyribose, and the hydration shell) is >17.5 eV for a given incident particle.^33^ These volumes are assumed to be filled with liquid water for physical track interactions using the physics list G4EmDNAPhysics_option8, used for consistency with the calculation of G‐values presented in this work.

For the DNA damage calculations, radicals undergo immediately the reactions (in Table III) with static DNA elements to produce indirect damage. We suppose that these reactions are totally diffusion‐controlled. Thus, the random reaction times between radicals and DNA elements are calculated by Eq. (1). A reaction between a radical and a DNA element is counted as a primary damage event. Thereafter, the radical is killed and the damaged DNA element is no longer available for further reaction. Note that our damage are primary damage events that are not necessarily transformed into single‐strand break (SSB) or double‐strand break (DSB). Going out of the voxel volume, the radical species will be discarded. Table III shows the reactions used between the $e_{\text{aq}}^{‐}$, H^•^, ^•^OH radicals and the DNA bases or 2‐deoxyribose and their reaction rates. For DNA damage calculations, we also perform a comparison with *SBS‐dynamic time step using 0.1 ps Minimum Time Steps* for overall virtual simulation time. This is *SBS‐dynamic time step using Minimum Time Steps of 0.1 ps*.

**Table III:** Reaction rates between the $e_{\text{aq}}^{‐}$, H^•^, ^•^OH radicals and the DNA bases or 2‐deoxyribose.^7^

Reaction	Reaction rate (10^9^ M^−1^ s^−1^)
2‐deoxyribose + ^•^OH	1.8
Adenine + ^•^OH	6.1
Guanine + ^•^OH	9.2
Thymine + ^•^OH	6.4
Cytosine + ^•^OH	6.1
2‐deoxyribose + $e_{\text{aq}}^{‐}$	0.01
Adenine + $e_{\text{aq}}^{‐}$	9.0
Guanine + $e_{\text{aq}}^{‐}$	14.0
Thymine + $e_{\text{aq}}^{‐}$	18.0
Cytosine + $e_{\text{aq}}^{‐}$	13.0
2‐deoxyribose + H^•^	0.029
Adenine + H^•^	0.10
Thymine + H^•^	0.57
Cytosine + H^•^	0.092

## 3 RESULTS AND DISCUSSION

### 3.A Results on radiolytic yields

First, the reliability and performance of our IRT method implementation in Geant4‐DNA are examined by using this method in the simulation to calculate time‐dependent G‐values of the chemical species created by water radiolysis, and comparing the results with other simulated data and experimental data from the literature. These quantities were calculated with our IRT method implementation (with both reaction schemes described in Section 2.B), the SBS‐dynamic time step of the current public release of Geant4‐DNA (Geant4 10.6), and the IRT method of TOPAS‐nBio code (Topas‐IRT).^23^ Note that the physical stage and physicochemical stage^31^ are the same for these three approaches.

**Fig. 3:**
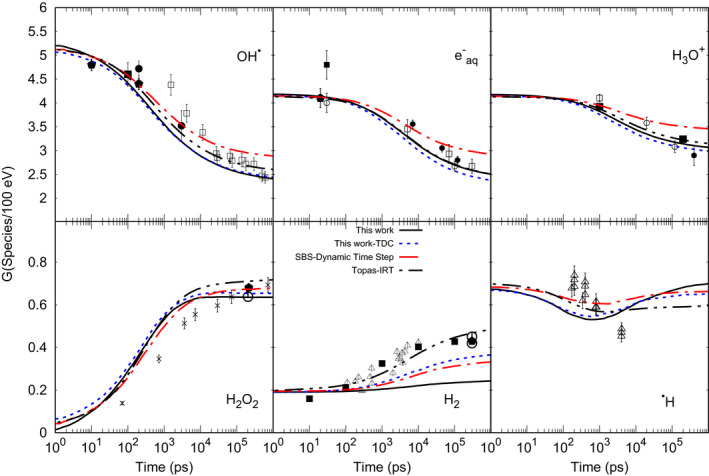
The G‐values as a function of time calculated by IRT (this work), Topas‐IRT, Geant4‐DNA‐SBS‐dynamic‐time‐step. Experimental data: OH^•^: ⎕ Laverne, 2000,^36^ ■ Jay‐Gerin and Ferradini, 2000,^37^ ● Jonah and Miller, 1977,^38^ El Omar et al., 2011.^39^ $e_{\text{aq}}^{‐}$: ⎕ Shiraishi et al., 1988,^40^ ■ Sumiyoshi and Katayama, 1982,^41^ ◯ Hunt et al., 1973^42^ and Wolff et al., 1973,^43^ ● Buxton, 1972,^44^ Muroya et al., 2005.^45^ H_3_O^+^: ⎕ Pikaev et al., 1977,^46^ ■ Cercek and Kongshaug, 1969,^47^ ◯ Anderson et al., 1985,^48^ ● Schmidt and Ander, 1969.^49^ H_2_O_2_: × Laverne, 2000,^36^ Elliot et al., 1993,^50^ ◯ Appleby and Schwarz, 1969.^51^ H_2_: ∆ Draganic and Draganic 1975,^52^ ■ LaVerne and Pimblott (1991),^53^ H^•^: ∆ Draganic and Draganic 1972.^54^

Figure 3 shows G‐values as a function of time for different species (^•^OH, H_3_O^+^, H^•^, $e_{\mathrm{aq}}^{‐}$, H_2_, and H_2_O_2_) produced by 80 keV electrons depositing a total energy of 1 keV. Our results follow the general trend of the experimental and calculated data. Our results are closer to Topas‐IRT than to the SBS‐dynamic time step results. Note that for both IRT simulations assigned, the same reaction types (see Section 2) and the reaction rates from Plante and Devroye^30^ are used, whereas SBS‐dynamic time step considers that all reactions are totally diffusion‐controlled. The differences about a few percent between our results and Topas‐IRT may be attributed to the re‐estimation of the random reaction times for each time step made by our method.

For each selected random reaction time, besides the calculation of the reactive products with the position approach (see Section 2.A.2), the position of all the other chemical species is also recalculated with the synchronous algorithm (Karamitros et al.^11^). In this way, both the evaluation of contact reactions and interaction with geometry can be performed (see Section 2.B.1). Nevertheless, the recalculated positions of chemical species forced to re‐evaluate the random reaction times which may in turn lead to a higher reactivity in comparison with TOPAS‐nBio, which implements the traditional IRT method. Our results show a difference of about 15% with SBS‐dynamic time step G values at 1 μs for *^•^OH*. Note that G‐value of *H_2_* shows an underestimation due to the inclusion of the spin effect. The balance material Eq. (11)12 was also verified, that is, the number of reduced species agreed with the number oxidative species within 0.23%, in the entire time domain.

(11) $$G\left(‐H_{2}O\right)=G\left(e_{\text{aq}}^{‐}\right)+2G\left(H_{2}\right)+G\left(H\right)=G\left({}^{\cdot }\text{OH}\right)+2G\left(H_{2}O_{2}\right).$$

### 3.B Results on damage yields

Damage yields presented here represent the addition of direct and indirect damage per particle to the DNA backbone and bases (see Table III). The Geant4‐DNA results of our IRT implementation were compared to Geant4‐DNA SBS public method by setting the parameters related to the time step (see Section 2): the current SBS‐dynamic time Step with varying time steps as shown in Table IV, the SBS‐dynamic time step with the same minimum time step value of 0.1 ps. For these time step models, the same parameters for diffusion coefficients and reaction rate coefficients, as available in Geant4‐DNA (see Table I), are used and the Brownian bridge technique is used.

**Table IV:** Variable minimum time steps proposed by Kreipl et al.^28^

Slice of simulated time (ps)	Time steps (ps)
1–10	0.1
10–100	1
100–10^3^	3
10^3^–10^4^	10
Above 10^4^	100

The starting point that motivated the developments around the IRT method is the reduction of the otherwise long computation times, as it is known to be much faster than SBS methods. Our implementation of the IRT, coupled with the synchronous algorithm, has resulted in a gain in calculation time for 500 keV protons and one voxel of complex DNA geometry by a factor of 30 relative to *SBS‐dynamic time step using Minimum Time Steps of 0.1 ps* and a factor of 15 relative to *SBS‐dynamic time step using variable Minimum Time Steps,* the latter currently the Geant4‐DNA default.

**Fig. 4:**
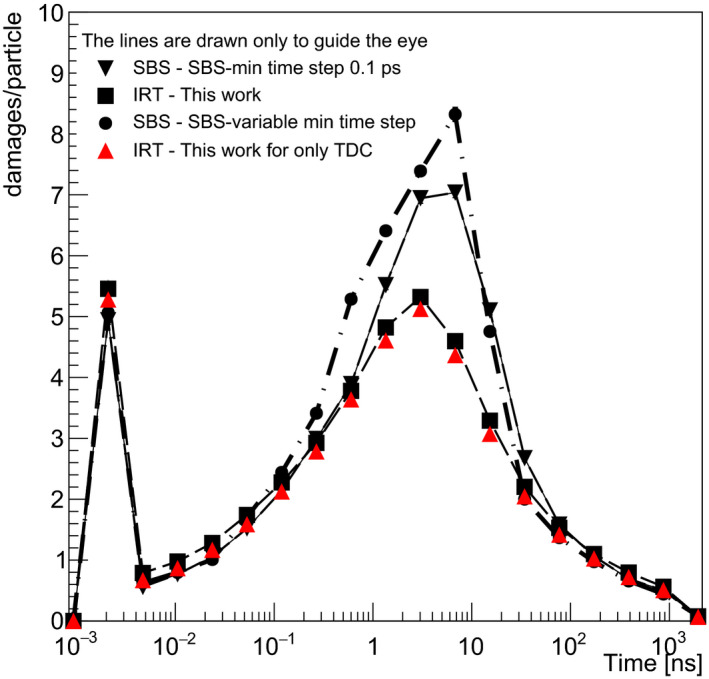
DNA damage yields (damages/particle) including direct (plotted at 2 ps) and indirect damage including DNA backbone and bases (second part of the curve starting from 4 ps) as a function of virtual time. Results are shown for SBS‐dynamic time step using 0.1 ps minimum time step (thin black line), SBS‐dynamic time step using minimum time step as a function of virtual time from Table IV (dash‐dot black line), IRT (dashed red line) from this work, and IRT using only totally diffusion‐controlled reaction are shown for comparison.

Figure 4 shows the damage yield produced per incident proton, at each value of the virtual time (not cumulated). For this calculations, 10^4^ initial protons of 500 keV were used traversing the voxel containing the chromatin fiber. Note that *SBS‐dynamic time step using Minimum Time Steps of 0.1 ps* is selected as the reference data in this study. While the first maximum shows the direct damage and indirect damage at 2 ps (therefore just right after the physicochemical stage), the second one shows the “active” reaction time of indirect damage of the simulation. We observe a reasonable agreement between the three models from 1 ps to 2 ns and from 20 to 100 ns. The discrepancies raise up in the range 2–20 ns between *SBS‐dynamic time step using Minimum Time Steps of 0.1 ps* and *SBS‐dynamic time step using variable Minimum Time Steps*. The latter shows a maximum difference of about 15% at 6 ns. This can be explained by inaccuracies in the counting of damage due to a too long minimum time step (10 ps in the range 1–10 ns).

For the IRT method (both schemes), we observe a difference of about 35% at 6 ns in comparison with *SBS‐dynamic time step of 0.1 ps*. The difference may be attributed to the higher reactivity of reactants and faster decrease than the SBS method for radical species as $e_{\mathrm{aq}}^{‐}$ *, H^•^, ^•^OH* over the virtual time that we observe, as well, by their G‐values in Fig. 3. As they participate in causing DNA damage (see Table III), their low concentration from 2 ns leads to the lower yield of DNA damage in comparison with *SBS‐dynamic time step of 0.1 ps*.

**Fig. 5:**
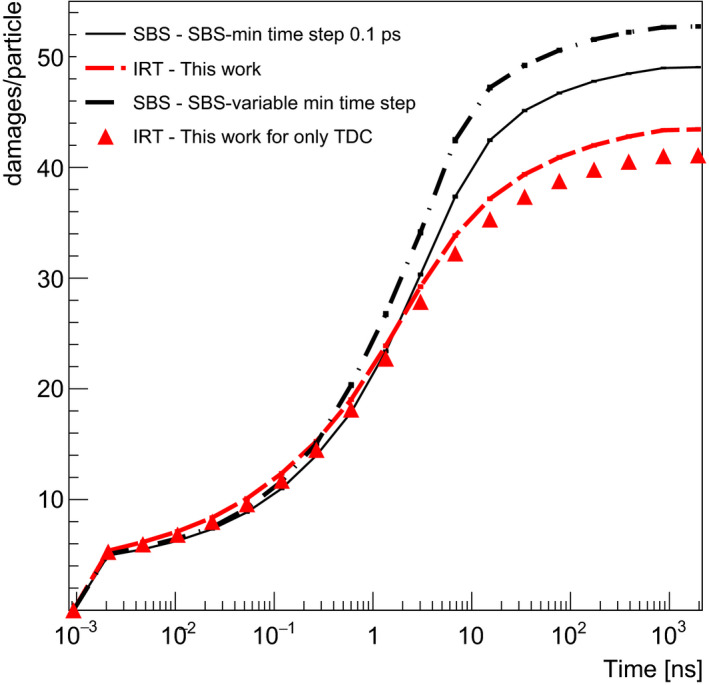
Cumulative damage yield as a function of virtual time corresponding to the results.

In order to compare the methods, we chose the total number of damages as the key quantity. Figure 5 shows the cumulative damage expressed in terms of damage per primary particle as a function of virtual time obtained by integrating the data of Fig. 4. Consequently, there is a good agreement of the cumulative damage between all methods for simulation times up to 3 ns. If we consider the IRT method we developed, the difference is of the order of 10% for higher simulation times due to the underestimation of reactions in the time range 2–20 ns as explained above. This difference is less than the uncertainty generally obtained in experimental DNA damage assessment some minutes after irradiation (as experimental data on indirect effects some ns after the irradiation are not possible to perform^34^).These results should be interpreted considering the fact that in concrete applications (e.g., cellular irradiations), the simulation time of the chemistry is an adjustable parameter set to reproduce scavenging effects that limit the diffusion of chemical species. For example, the simulation time of the chemical step with the SBS method of Geant4‐DNA was limited to 1 ns for the calculation of damage to a chromatin fiber^33^ or 2.5 ns for damage to a complete cell nucleus.^2^, ^33^ In the latter case, it was suggested that a time of 10 ns would be more appropriate.^35^ Under these conditions, it must therefore be considered that the IRT method presented in this study gives results that are reasonably close to those of the method we have considered as a reference for Geant4‐DNA calculations about 10% at 10 ns (SBS‐dynamic time step using 0.1 ps minimum user time step).

## 4 CONCLUSIONS

The IRT‐based time step method developed in this work was successfully implemented in the chemistry module of Geant4‐DNA. This implementation allows calculation of the positions of the particles at each time step. It may be used in the simulations of complex distributions of molecules and radiolytic species that evolve with time as DNA is damaged. The development succeeded in our objective: keeping the efficiency in computation time of the IRT method while taking into account the constraints related to the calculation of DNA damage by having access to the position of reactants in the DNA geometrical frame. The obtained G‐values as function of virtual time for *^•^*OH, $e_{\text{aq}}^{‐}$, H_2_, and H_2_O_2_ produced by 80 keV electron tracks are in generally good agreement with experimental data, considering the experimental uncertainty, and with results from IRT and SBS simulations, validating the developed method. By using a piece of heterochromatin fiber, consisting of 3640 nucleotide pairs, we have demonstrated the use of our IRT implementation in a complex DNA geometry. We observe that IRT shows a difference of about 35% decrease in DNA damage yields at 6 ns in comparison with SBS‐dynamic time step (*Minimum User Time Step* of 0.1 ps). Despite this difference, the total number of damages calculated over a typical simulation time of the chemical part not exceeding 10 ns agrees within 10% with the *SBS‐dynamic time step* method currently available in Geant4‐DNA. This difference is acceptable, considering the uncertainty in the SBS‐dynamic time step results and the large gain in computational time of a factor of 30. This gain is particularly valuable for radiobiology applications. In the near future, the potential of the simulation of the chemical step of Geant4‐DNA will be extended not only by the addition of this IRT variant but also by the addition of the traditional IRT method made available by the Geant4‐DNA collaboration. In this frame, two other IRT versions have been developed to cover several types of applications and will be released after their publication. Investigations on the conditions of validity of each method and comparisons should be made available to allow Geant4‐DNA users to choose, improve, or propose the method that best suits their needs. A comparison of simulations with experimental data on plasmid geometries could be a first step.

## ACKNOWLEDGEMENT

JRM and BF were supported by the National Institutes of Health/National Cancer Institute (NIH/NCI grant no. R01 CA187003: ‘‘TOPAS‐nBio: A Monte Carlo tool for radiation biology research’’).
